# Prediction of bone metastasis in non-small cell lung cancer based on machine learning

**DOI:** 10.3389/fonc.2022.1054300

**Published:** 2023-01-09

**Authors:** Meng-Pan Li, Wen-Cai Liu, Bo-Lin Sun, Nan-Shan Zhong, Zhi-Li Liu, Shan-Hu Huang, Zhi-Hong Zhang, Jia-Ming Liu

**Affiliations:** ^1^ Department of Orthopedic Surgery, The First Affiliated Hospital of Nanchang University, Nanchang, China; ^2^ The First Clinical Medical College of Nanchang University, Nanchang, China; ^3^ Department of Orthopaedics, Shanghai Jiao Tong University Affiliated Sixth People's Hospital, Shanghai, China; ^4^ Institute of Spine and Spinal Cord, Nanchang University, Nanchang, China

**Keywords:** bone metastasis, NSCLC, machine learning, XGB algorithm, SEER

## Abstract

**Objective:**

The purpose of this paper was to develop a machine learning algorithm with good performance in predicting bone metastasis (BM) in non-small cell lung cancer (NSCLC) and establish a simple web predictor based on the algorithm.

**Methods:**

Patients who diagnosed with NSCLC between 2010 and 2018 in the Surveillance, Epidemiology and End Results (SEER) database were involved. To increase the extensibility of the research, data of patients who first diagnosed with NSCLC at the First Affiliated Hospital of Nanchang University between January 2007 and December 2016 were also included in this study. Independent risk factors for BM in NSCLC were screened by univariate and multivariate logistic regression. At this basis, we chose six commonly machine learning algorithms to build predictive models, including Logistic Regression (LR), Decision tree (DT), Random Forest (RF), Gradient Boosting Machine (GBM), Naive Bayes classifiers (NBC) and eXtreme gradient boosting (XGB). Then, the best model was identified to build the web-predictor for predicting BM of NSCLC patients. Finally, area under receiver operating characteristic curve (AUC), accuracy, sensitivity and specificity were used to evaluate the performance of these models.

**Results:**

A total of 50581 NSCLC patients were included in this study, and 5087(10.06%) of them developed BM. The sex, grade, laterality, histology, T stage, N stage, and chemotherapy were independent risk factors for NSCLC. Of these six models, the machine learning model built by the XGB algorithm performed best in both internal and external data setting validation, with AUC scores of 0.808 and 0.841, respectively. Then, the XGB algorithm was used to build a web predictor of BM from NSCLC.

**Conclusion:**

This study developed a web predictor based XGB algorithm for predicting the risk of BM in NSCLC patients, which may assist doctors for clinical decision making

## Introduction

Lung cancer, as one of the most common malignant tumors worldwide, has an annual incidence of 2 million and causes 1.76 million deaths each year (1). Non-small cell lung cancer (NSCLC) accounts for approximately 85% of lung cancer cases, which has an improving overall survival rate due to better therapy (2). However, bone metastasis (BM) is a negative prognostic factor in NSCLC patients. Studies have reported that the incidence of BM in patients with NSCLC is 26-36%, and the 2-year survival rate of patients with BM is 3% (3). Also, bone metastases often result in a range of complications, such as pain, hypercalcemia, spinal cord compression, pathological fractures and neurological defect, which will decrease the patient’s quality of life (4).

Early diagnosis and intervention in patients with BM could significantly improve prognosis of patients. At present, bone scan is the most classic way to detect bone related diseases (5). But due to its low sensitivity to bone metastases, early BM from cancer is often not detected. Although studies had shown that PET-CT can improve the detection rate of small bone lesions (6), it is limited as a screening tool due to its high cost and high radiation. Therefore, bone scans and PET-CT are recommended only when there is a suspicious bone-related event, which usually occurs 5 months after BM (7). By then, many NSCLC patients may have developed multiple metastases, and the prognosis of patients is poor.

Previous studies (8–10) reported that histopathological type, gender, histological differentiation, serum CA-125, ALP, and multiple lymph node metastasis are independent risk factors for BM of lung cancer, which lays the foundation for prediction model construction. Zhang Chao et al (10). constructed a nomogram to predict the BM in different histological types of lung cancer based on the traditional logistic model. However, the limitations of this method in prediction accuracy and processing big data have made it difficult to make great breakthroughs in precision medicine (11, 12). Therefore, advanced machine learning models were used in this study.

Compared with traditional logistical model, machine learning (ML) technology can unlock more information in large datasets to achieve the purpose of outcome prediction and have higher accuracy (12). There already are many applications of this technology throughout science and society ranging from driverless cars to Board games to decision-making (12, 13). In biomedicine, the development of big data in healthcare (14, 15) offers great potential for ML to understand disease and health and ML has been used in clinical diagnostics, precision therapeutics, and health monitoring (16).

Therefore, in this study, we aimed to found a machine learning algorithm with good prediction performance, and establish it as a web-based calculator that can be easily used to predict the risk of BM in NSCLC patients.

## Methods

### Study population

**Figure 1:**
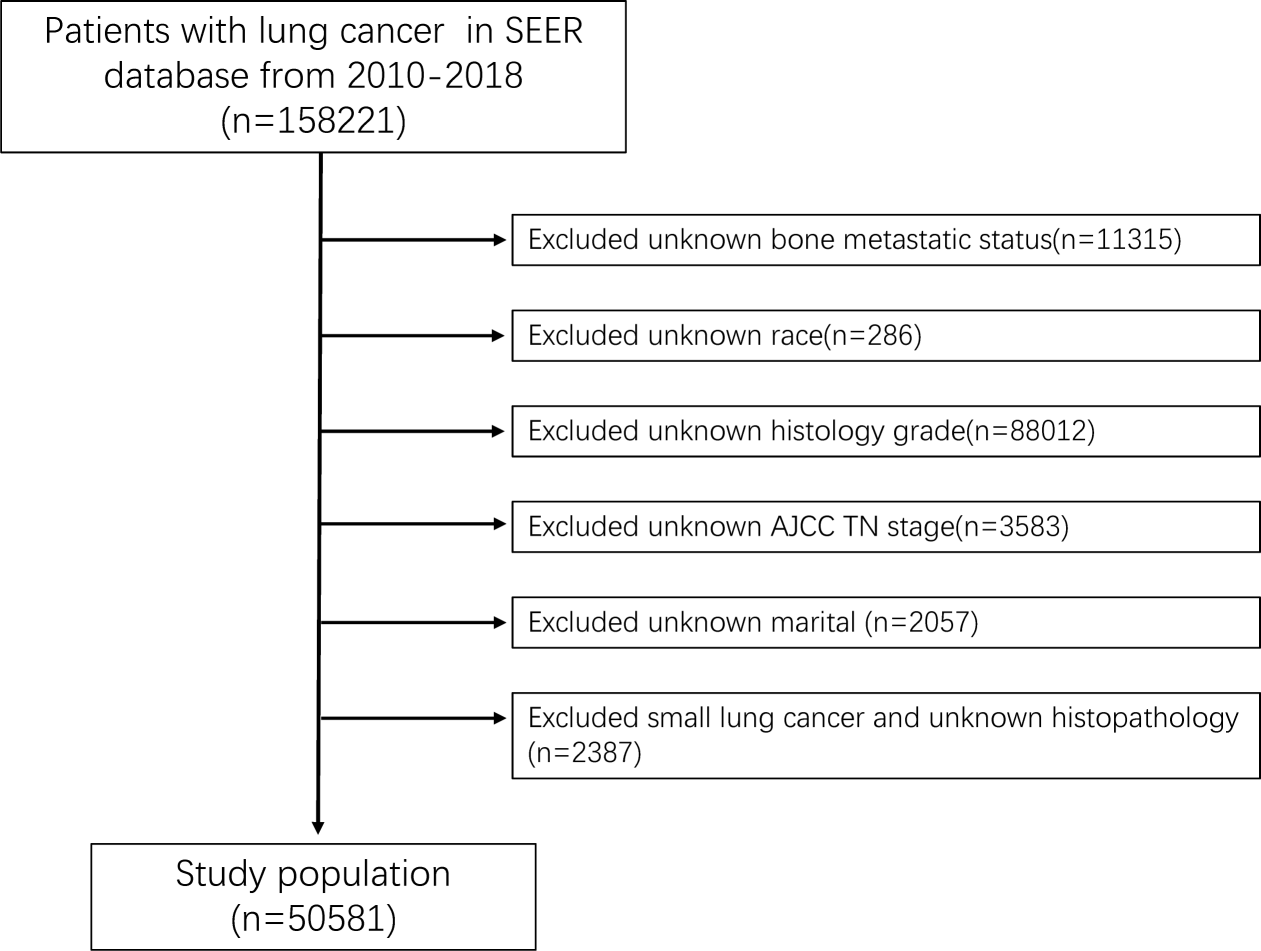
The study flow chart of case screening.

In the study, we used SEER*stat 8.4.0 software to download the patients’ data from the SEER-Medicare database submitted in November 2020. Patients diagnosed with lung cancer between 2010 and 2018 were involved in this study. Exclusion criteria were detailed as follows: (1) The histological type of lung cancer is small cell cancer and unknown; (2) The information of T stage, N stage, race, grade, marital status, and bone metastatic status missed or unknown; (3) LC is not the first tumor. A study flow chart of case screening was presented in **Figure 1**. Additionally, 507 patients newly diagnosed with NSCLC were included in this study between January 2007 and December 2016 at the First Affiliated Hospital of Nanchang University.

### Data selection

In this study, 11 variables related to the clinicopathology and demographics of patients were selected for analysis. Demographic variables included race, sex, marital and age. Clinicopathological variables included primary site, grade, histology, T stage, N stage, laterality and chemotherapy. According to the ICD-O-3 codes, histological types of NSCLC were divided into 5 categories, including adenocarcinoma (814-838), squamous cell carcinoma (805-808), adenosquamous carcinoma (856), large cell carcinoma (8012-8014) and others. All NSCLC patients were staged according the AJCC 7th edition guidelines and SEER staging information. In addition, we divided patients into two groups at 60 years to analyze the effect of age on outcome events by referring to the study of Zhou et al (8).

### Data pre-processing and feature engineering

All statistical analyses were conducted with Python3.8, SPSS 23 and R 4.2.0. In this study We performed a logistic regression analysis on data collected in the SEER database to identify suitable variables for machine learning model by using SPSS 23 software. Significant variables between BM and non-BM patients were identified by univariate logistic regression analysis (P<0.05). Then, these variables were enclosed within multivariate logistic regression analysis, and variables with a P < 0.05 in multivariate logistic regression analysis were subjected for further analysis of ML model. Correlation analysis was used to analyze the correlation among the selected features. Since this data set is an unbalanced data set, the over-sampling method were adopted for data processing (17). The key of this method is to oversampling the data samples of small classes to increase the number of data samples of small classes to improve the accuracy of the model.

For the 507 external samples, the cancer stage and grade were unified according to the AJCC 7th edition criteria, so that the parameters of the two data sets could be matched. Missing values were imputed by R mouse package using classification and regression tree principle (18). Meanwhile, to compare the importance of each feature, we extract the feature importance of each variable in the machine learning model according to the Permutation Importance principle (19).

### Model establishment and evaluation

During the modeling establishment, SEER data set was used as internal data to build and validate the models, while hospital data were used as external validation data to validate and evaluate the predictive ability of the machine learning models. The risk stratification threshold of the model was set to 0.5(50%) (20).

Six commonly used classifier algorithms were chosen to this study, including three ensemble algorithms (12) (Random Forest (RF), Gradient Boosting Machine (GBM), eXtreme gradient boosting (XGB)) and three simple classification algorithms (Logistic Regression (LR), Decision tree (DT), Naive Bayes classifiers (NBC)). The ML models were trained using Python software. In the internal test, all SEER data were divided into 10 parts for 10-fold cross-validation (21). For external testing, external data was imported directly into the built model for verification. The area under the receiver operating characteristic curve (AUC), sensitivity, specificity, accuracy and F-score were evaluated indicators of ML algorithms. Comparing the evaluation indexes of each model, the best model was selected to build a network predictor.

## Results

### Demographic and pathological characteristics

In this study, 50581 patients diagnosed with NSCLC in the SEER database were included. Of whom, 5087 (10.06%) developed BM and 45494 (89.94%) had no BM. Meanwhile, 507 NSCLC patients at the First Affiliated Hospital of Nanchang University were selected for external validation of the models. The Characteristics of all data is showed in **Table 1**.

**Table 1:** Clinical and pathological characteristics of training set and external test set.

Variables	Train Set (n=50581)		External Test Set (n=507)	
	NBM (%) *N=*45494	BM (%) *N=*5087	NBM (%) *N=*441	BM (%) *N=66*
Race:				
Black	4853 (10.7%)	628 (12.3%)	0	0
White	36818 (80.9%)	3924 (77.1%)	0	0
Others	3823 (8.40%)	535 (10.5%)	441 (100%)	66 (100%)
Sex:				
Female	22809 (50.1%)	2189 (43.0%)	206 (46.7%)	28 (42.4%)
Male	22685 (49.9%)	2898 (57.0%)	235 (53.3%)	38 (57.6%)
Primary.Site:				
Main bronchus	1097 (2.41%)	200 (3.93%)	13 (2.95%)	5 (7.58%)
Upper lobe	25860 (56.8%)	2760 (54.3%)	247 (56.0%)	42 (63.6%)
Middle lobe	2372 (5.21%)	246 (4.84%)	32 (7.26%)	3 (4.55%)
Lower lobe	14160 (31.1%)	1434 (28.2%)	137 (31.1%)	14 (21.2%)
Others	2005 (4.41%)	447 (8.79%)	12 (2.72%)	2 (3.03%)
Grade:				
Grade I	7090 (15.6%)	302 (5.94%)	113 (25.6%)	5 (7.58%)
Grade II	17875 (39.3%)	1408 (27.7%)	177 (40.1%)	24 (36.4%)
Grade III	19567 (43.0%)	3217 (63.2%)	148 (33.6%)	35 (53.0%)
Grade IV	962 (2.11%)	160 (3.15%)	3 (0.68%)	2 (3.03%)
Laterality:				
Left	18798 (41.3%)	2129 (41.9%)	164 (37.2%)	29 (43.9%)
Right	26393 (58.0%)	2855 (56.1%)	275 (62.4%)	36 (54.5%)
Others	303 (0.67%)	103 (2.02%)	2 (0.45%)	1 (1.52%)
Histology:				
Adenocarcinoma	24098 (53.0%)	3113 (61.2%)	312 (70.7%)	42 (63.6%)
squamous cell carcinoma	14106 (31.0%)	1033 (20.3%)	75 (17.0%)	9 (13.6%)
Adenosquamous carcinoma	696 (1.53%)	65 (1.28%)	9 (2.04%)	1 (1.52%)
Large cell carcinoma	668 (1.47%)	89 (1.75%)	2 (0.45%)	1 (1.52%)
Others	5926 (13.0%)	787 (15.5%)	43 (9.75%)	13 (19.7%)
T stage:				
T1	15875 (34.9%)	483 (9.49%)	156 (35.4%)	3 (4.55%)
T2	14885 (32.7%)	1359 (26.7%)	146 (33.1%)	11 (16.7%)
T3	7984 (17.5%)	1365 (26.8%)	82 (18.6%)	13 (19.7%)
T4	6750 (14.8%)	1880 (37.0%)	57 (12.9%)	39 (59.1%)
N stage:				
N0	27907 (61.3%)	1027 (20.2%)	269 (61.0%)	13 (19.7%)
N1	4431 (9.74%)	482 (9.48%)	36 (8.16%)	5 (7.58%)
N2	10121 (22.2%)	2389 (47.0%)	106 (24.0%)	35 (53.0%)
N3	3035 (6.67%)	1189 (23.4%)	30 (6.80%)	13 (19.7%)
Chemotherapy:				
NO	29077 (63.9%)	2110 (41.5%)	274 (62.1%)	23 (34.8%)
YES	16417 (36.1%)	2977 (58.5%)	167 (37.9%)	43 (65.2%)
Marital				
Unmarride	12987 (28.5%)	1504 (29.6%)	–	–
Married	32507 (71.5%)	3583 (70.4%)	–	–
Age:				
<=60	8944 (19.7%)	1260 (24.8%)	79 (17.9%)	20 (30.3%)
>60	36550 (80.3%)	3827 (75.2%)	362 (82.1%)	46 (69.7%)

BM, bone metastasis; NBM, no bone metastasis.

### Logistic regression analysis

The univariate analysis based on the training set is presented in **Table 2**, and the results showed that marital was not significantly associated with the BM in NSCLC patients (*P* > 0.05). The remaining ten significant variables were selected for multivariate logistic regression analysis. Multivariate logistic regression analysis (**Table 2**) indicated that seven variables, including sex, grade, laterality, histology, T stage, N stage, and chemotherapy, were independent risk factors for BM of NSCLC. The seven variables were used further machine learning model study.

**Table 2:** Univariate analysis and multivariate logistic regression analysis of variables.

Variables	Univariate Analysis		Multivariate Logistic Analysis	
	OR (95%Cl)	P-value	OR (95%Cl)	P-value
Race:				
Black	Reference		Reference	
White	0.824 (0.753-0.901)	<0.001	0.976 (0.887-1.074)	0.617
Others	1.081 (0.956-1.223)	0.212	1.082 (0.948-1.234)	0.242
Sex:				
Female	Reference		Reference	
Male	1.331 (1.256-1.411)	<0.001	1.221 (1.147-1.301)	<0.001
Primary.Site:				
Main bronchus	Reference		Reference	
Upper lobe	0.585 (0.501-0.684)	<0.001	1.036 (0.877-1.222)	0.679
Middle lobe	0.569 (0.466-0.695)	<0.001	1.160 (0.936-1.439)	0.175
Lower lobe	0.555 (0.473-0.652)	<0.001	0.148 (0.967-1.362)	0.115
Others	1.223 (1.019-1.467)	0.031	1.206 (0.990-1.469)	0.062
Grade:				
Grade I	Reference		Reference	
Grade II	1.849 (1.628-2.100)	<0.001	1.434 (1.254-1.640)	<0.001
Grade III	3.860 (3.420-4.356)	<0.001	1.888 (1.658-2.150)	<0.001
Grade IV	3.905 (3.187-4.784)	<0.001	1.666 (1.333-2.084)	<0.001
Laterality:				
Left	reference		Reference	
Right	0.955 (0.900-1.013)	0.128	0.912 (0.855-0.972)	0.005
Others	3.001 (2.390-3.770)	<0.001	1.227 (0.944-1.595)	0.126
Histology:				
Adenocarcinoma	Reference		Reference	
squamous cell carcinoma	0.567 (0.527-0.610)	<0.001	0.428 (0.396-0.463)	<0.001
Adenosquamous carcinoma	0.723 (0.559-0.935)	0.013	0.587 (0.449-0.769)	<0.001
Large cell carcinoma	1.031 (0.824-1.291)	0.787	0.729 (0.569-0.932)	0.012
Others	1.028 (0.946-1.117)	0.515	0.875 (0.799-0.957)	0.004
T stage:				
T1	Reference		Reference	
T2	3.001 (2.698-3.337)	<0.001	1.986 (1.778-2.219)	<0.001
T3	5.619 (5.048-6.255)	<0.001	3.007 (2.682-3.371)	<0.001
T4	9.154 (8.250-10.157)	<0.001	4.029 (3.596-4.513)	<0.001
N stage:				
N0	Reference		Reference	
N1	2.956 (2.641-3.309)	<0.001	2.163 (1.923-2.433)	<0.001
N2	6.414 (5.941-6.925)	<0.001	3.988 (3.664-4.341)	<0.001
N3	10.645 (9.715-11.666)	<0.001	5.700 (5.152-6.307)	<0.001
Chemotherapy:				
NO	Reference		Reference	
YES	2.499 (2.356-2.651)	<0.001	1.108 (1.037-1.184)	<0.001
Marital				
Unmarried	Reference			
Married	0.952 (0.893-1.014)	0.127		
Age:				
<=60	Reference		Reference	
>60	0.743 (0.695-0.795)	<0.001	0.992 (0.922-1.068)	0.837

OR, odds ratio.

### Correlation analysis of features

**Figure 2:**
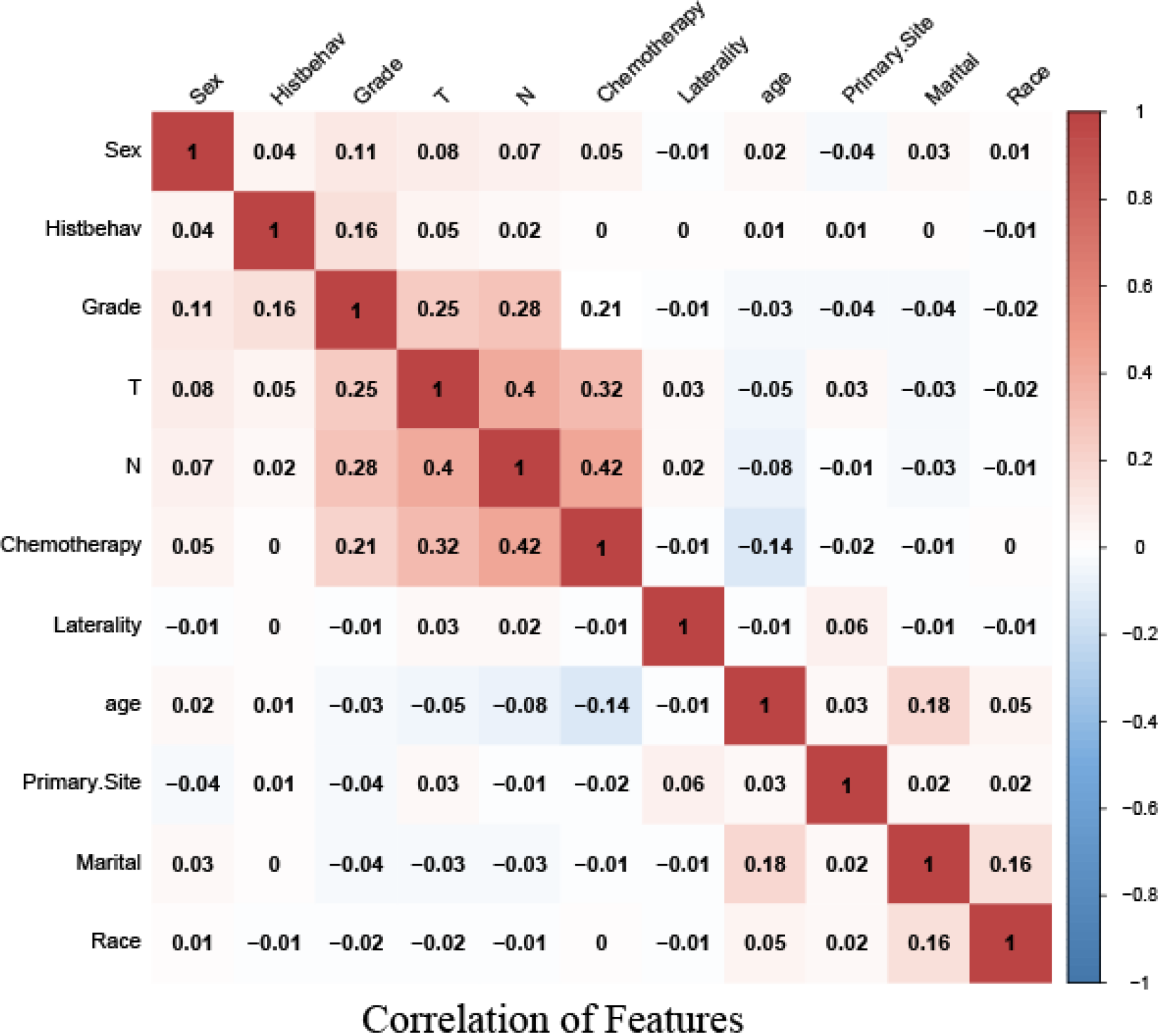
Heat map of the correlation of features.

Correlation analysis between data features is often used to measure the degree of correlation between factors. To identify the independence between features, we obtained a correlation heat map by Spearman correlation analysis. The figure showed that there was no strong correlation among these 11 features (**Figure 2**).

### Importance of features on prediction

**Figure 3:**
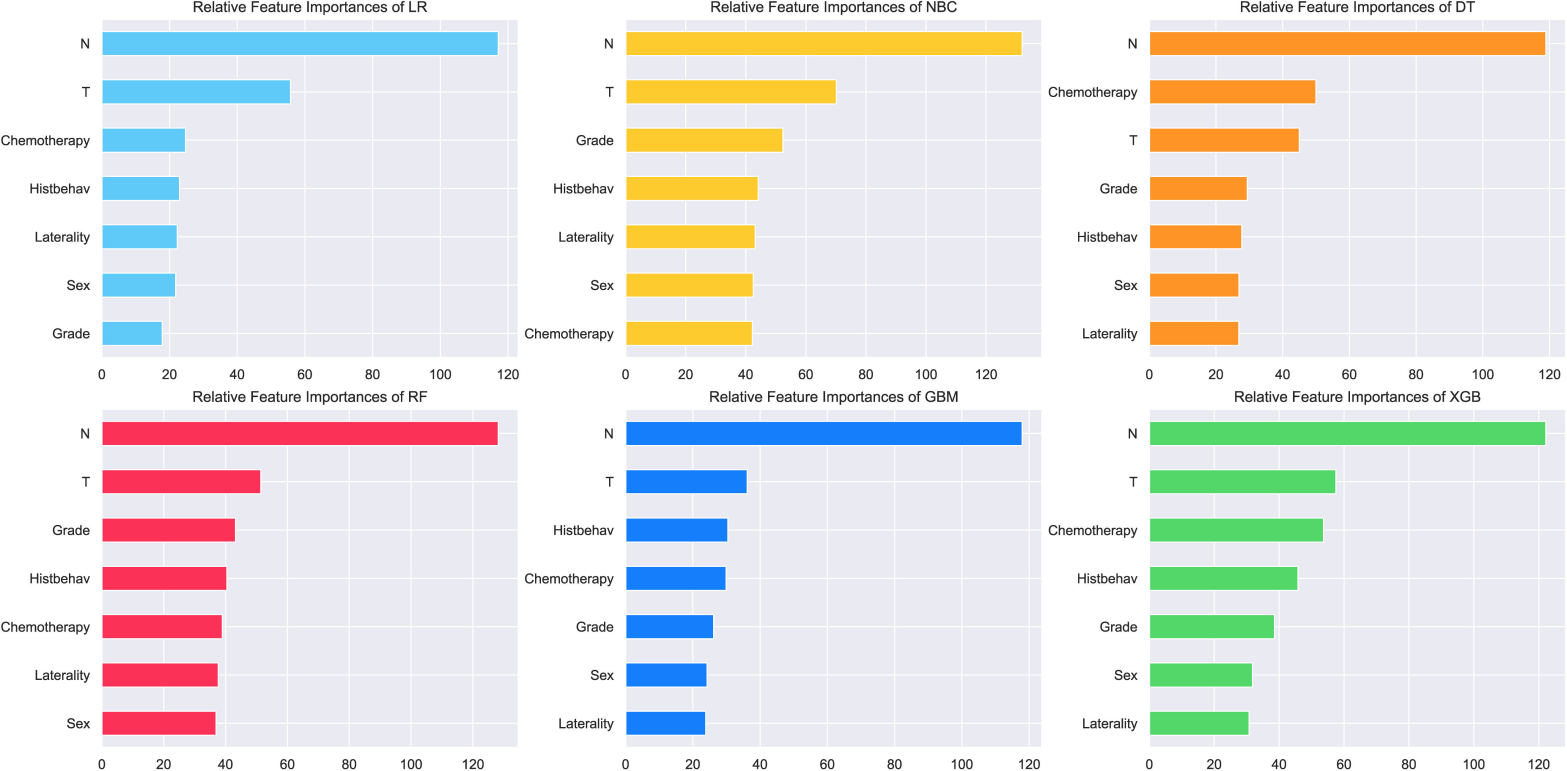
Feature importance of different models.

The importance of features extracted from each machine learning algorithm are shown in **Figure 3**. Variables screened by univariate and multivariate logistic analysis all have made extraordinary contributions to prediction in the six models. N-stage ranked top one in feature importance of all prediction models, indicating that N-stage has a great influence on BM of NSCLC, followed by T-stage. In most algorithms, grading, histology, laterality, gender and chemotherapy ranked the last five, with no significant difference in their contributions to the model. N-stage, T-stage, chemotherapy, histology, grade, sex and laterality are arranged in descending order in XGB model.

### Model performance

**Figure 4:**
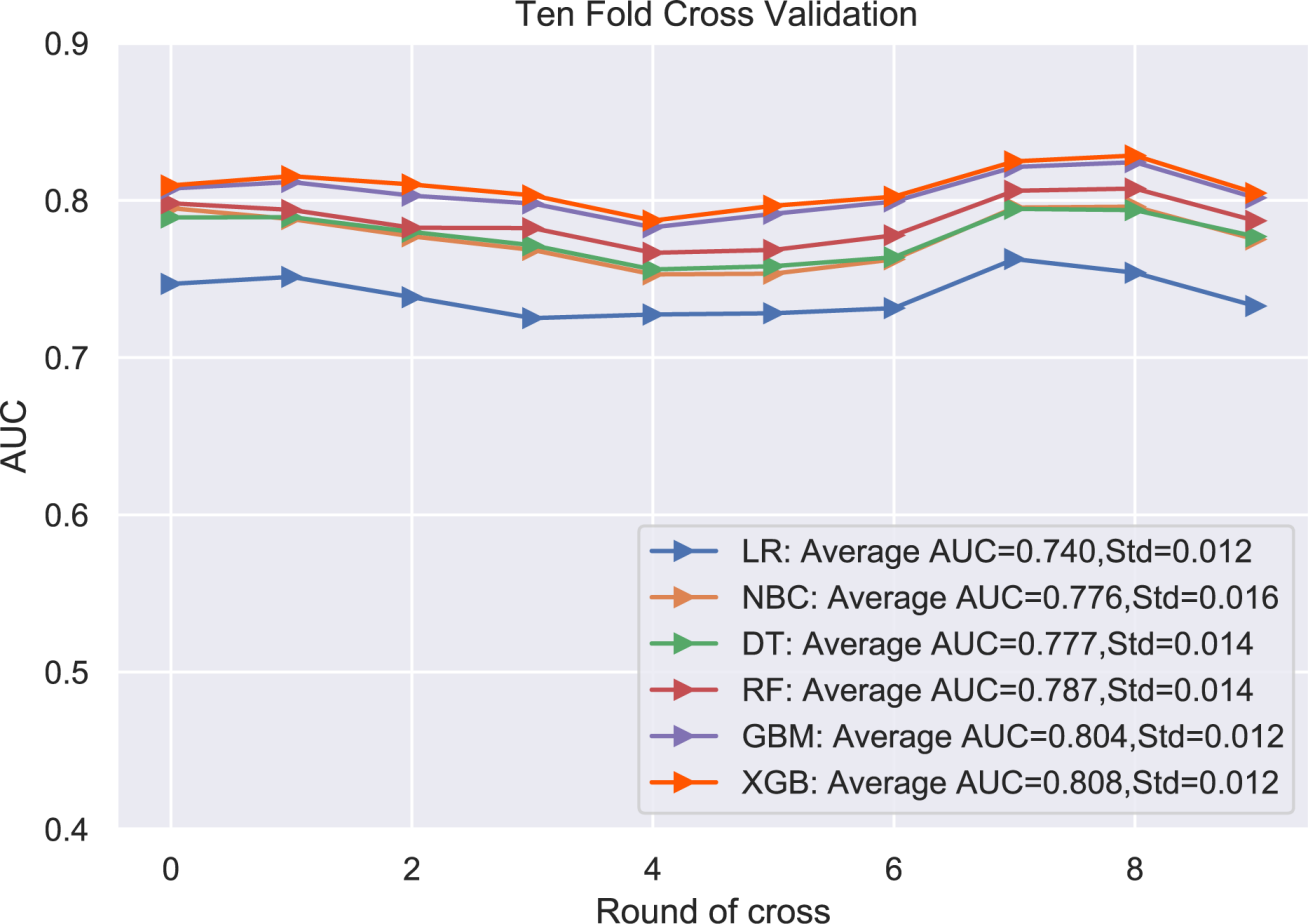
Ten-fold cross-validation results of different machine learning models.

**Figure 5:**
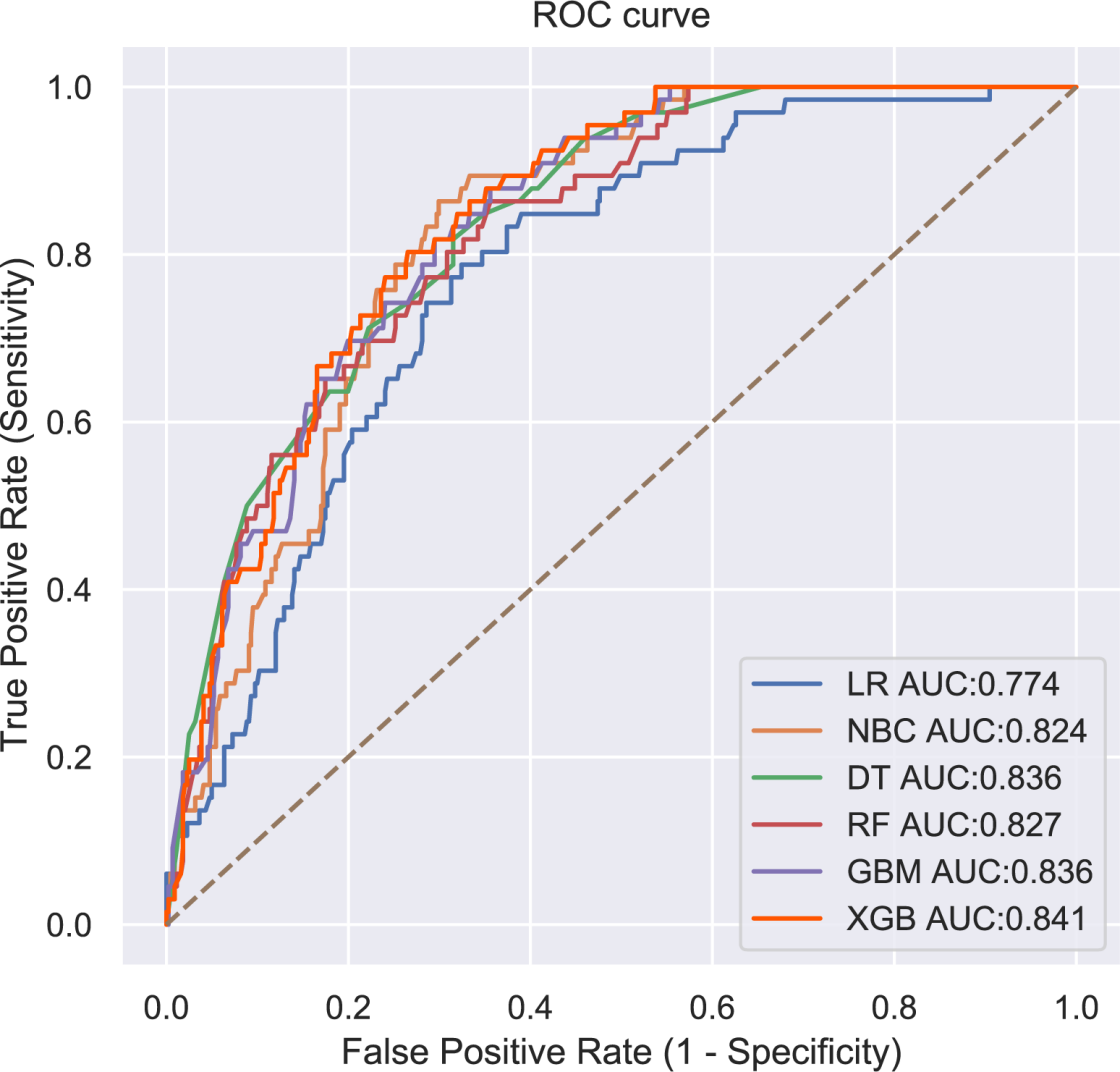
The roc curves of different machine learning models in external test set.

**Figure 6:**
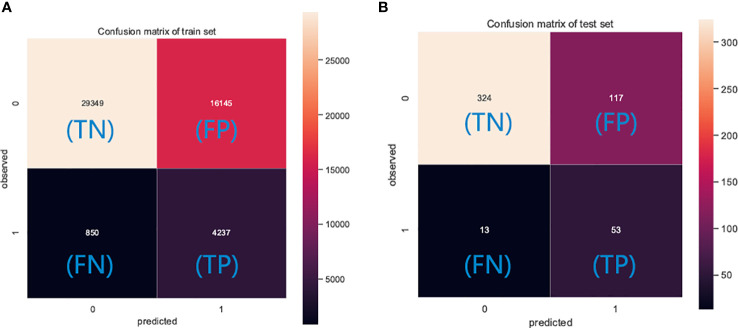
The confusion matrix of the XGB model in the **(A)** training set and the **(B)** test set. TP, true positive; TN, true negative; FP, false positive; FN, false negative.

The performance of the six predictive models is described in **Figures 4**, **5** and **Table 3**. Internal ten-fold cross-validation (**Figure 4**) showed that XGB model performed best among the six models with an average AUC of 0.808, followed by the GBM model (AUC=0.804). External test validation was shown in **Table 3** and **Figure 5**. Interestingly, the XGB model also achieves the best AUC score (0.841) in the external test validation and the score of accuracy, sensitivity (recall rate) and specificity were 0.744, 0.735 and 0.803, respectively. The confusion matrix (**Figure 6**) of the XGB model in the training set and the test set indicated its high accuracy.

**Table 3:** Prediction performance of different models.

	LR	NBC	DT	RF	GBM	XGB
Auc	0.774	0.824	0.836	0.827	0.836	0.841
Accuracy	0.694	0.738	0.698	0.734	0.720	0.744
Sensitivity	0.687	0.728	0.685	0.732	0.705	0.735
Specificity	0.742	0.803	0.788	0.742	0.818	0.803
F-score	0.387	0.444	0.405	0.420	0.432	0.449

LR, logistic regression; NBC, Naive Bayes classifiers; DT, decision tree; RF, random forest; GBM, Gradient Boosting Machine; XGB, eXtreme gradient boosting.

### Web predictor

**Figure 7:**
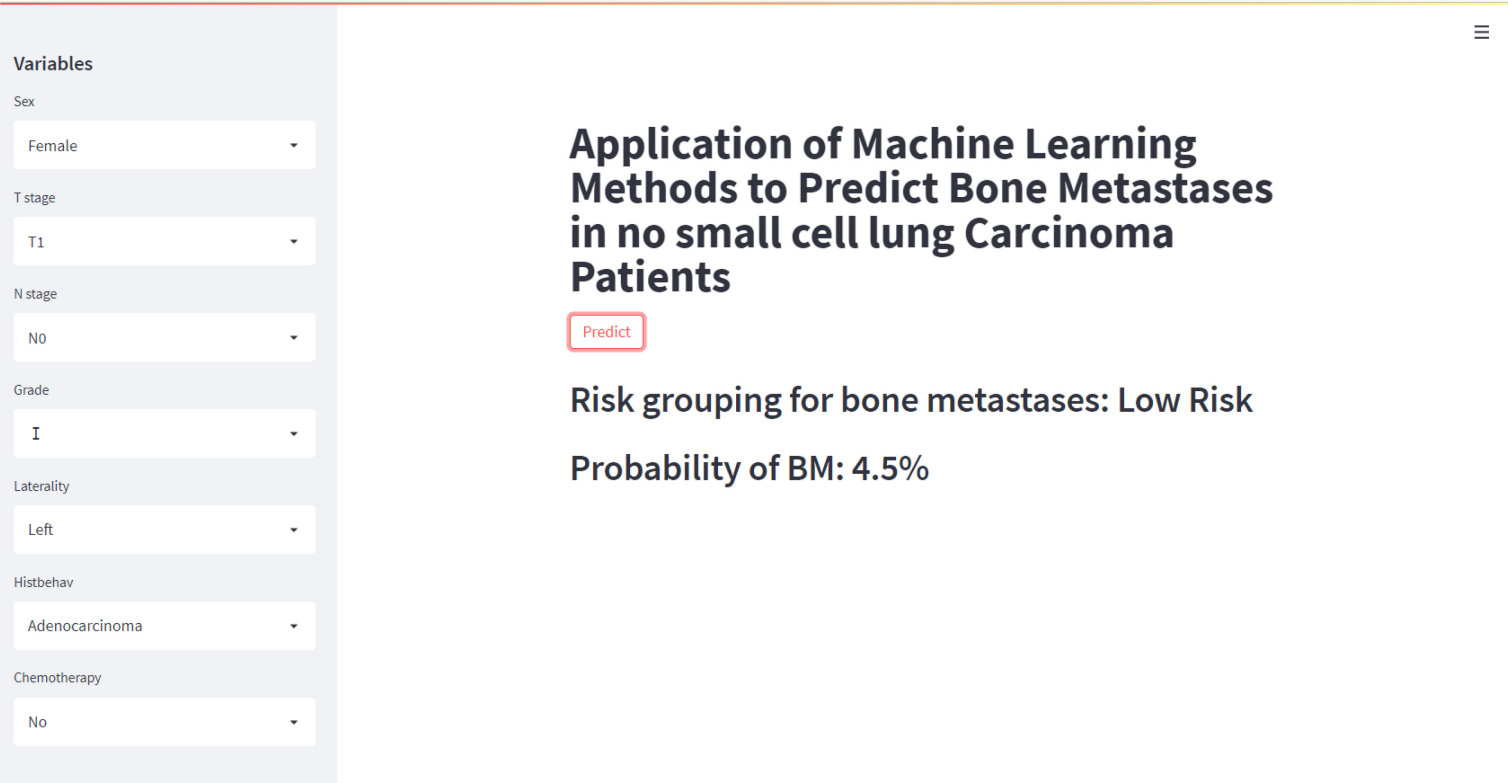
A web predictor for predicting bone metastases in no small cell lung carcinoma patients.

In this study, a web predictor based on the XGB model, which has the best predictive performance on BM of NSCLC patients, was developed to assist doctors to make more accurate clinical decisions. The odds of BM from NSCLC patients could be easily calculated by simply setting the variables associated with BM given on the web predictor. (https://share.streamlit.io/liuwencai6/lung_bone/main/lung.py) (**Figure 7**). When the predicted value of the patient is greater than 0.5, the network predictor shows high risk.

## Discussion

**Figure 8:**
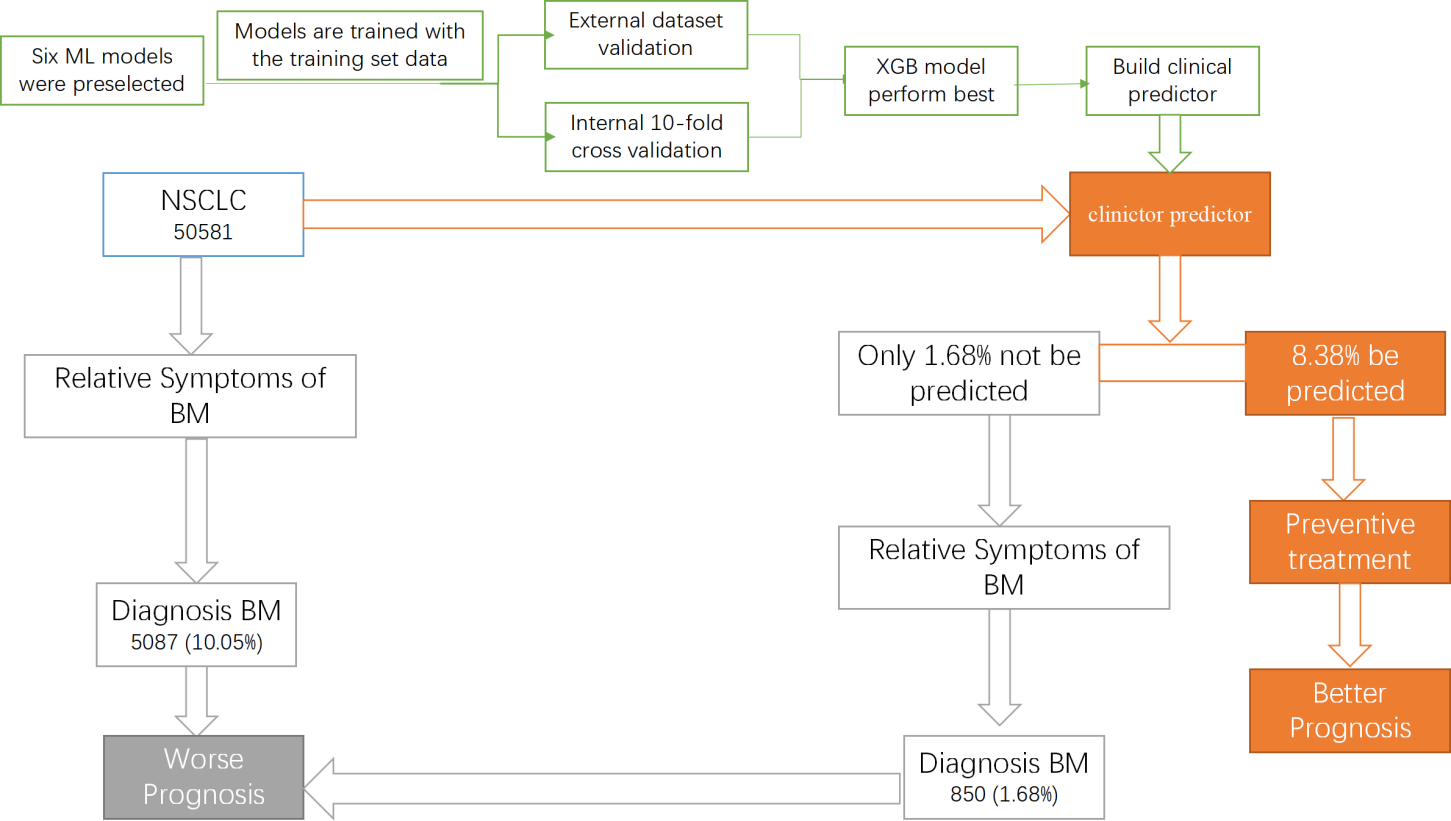
The construction process of the clinical predictor and the outcomes of the NSCLC with and without the predictor.

Lung cancer is one of the most common malignant tumors, which has an annual incidence of 2 million (1). Traditionally, lung cancer is divided into two types, including small cell lung cancer and NSCLC, with NSCLC accounting for 85% of cases (22). Compared with small cell carcinoma, NSCLC patients often has a better prognosis because of its slow growth and division (23). However, BM is considered an essential risk factor for the prognosis of NSCLC (24). Studies have reported that the incidence of BM in patients with NSCLC is 26-36% (3). However, the Lung Cancer National Comprehensive Cancer Network (NCCN) screening guidelines do not recommend routine bone imaging evaluation in asymptomatic patients (10). In order to identify lung cancer patients with high risk of BM, we innovatively constructed a clinical predictor based on an advanced ML algorithm (XGB). **Figure 8** shows the construction process of the clinical predictor and the outcomes of the NSCLC with and without the predictor.

Artificial intelligence (AI) is a field of research in which computers are applied to mimic human intelligence which has been successfully applied in various fields, including driverless driving, face recognition and music creation (25–27). ML is a subfield of AI that focuses on developing algorithms to learn from data (11). Therefore, the emergence of electronic medical records (28) (EMR) has created a huge amount of analyzable data in the medical field, which provides the potential for the development of ML in the medical field. In biomedicine, there have been many studies using ML algorithms to guide clinical diagnosis and treatment, Including COVID-19 and cancer metastases field (19, 29). Statistical review of ML in medical field by Kaustubh Arun Bhavsar et al. suggested that ML techniques can help clinicians make better clinical decisions and improve patient care and overall health (30).

In this study, we compared the performance of six different algorithms and found that XGB algorithm perform best. XGB algorithm is an efficient, flexible and scalable machine learning algorithm classifier that has been widely used in the medical filed, such as COVID-19, Chronic Kidney Disease Diagnosis and BM of prostate cancer (PCa) (19, 31, 32). Liu et al (19) compared the diagnostic ability of six algorithm models, including XGB, to predict BM of PCa, and found that XGB model performed best. The consistency of the results indicates that the XGB algorithm has great potential in medical applications. Compared with the traditional logistic regression model, XGB model can process big data efficiently and has higher accuracy (33). **Table 4** shows the strengths and weaknesses of the previous model as well as the proposed model (33).

**Table 4:** The strength and weakness of the previous model and the proposed model.

	Strength	Weakness
XGB model	Regularization prevents overfitting High precision Evaluating feature importance Automatically handle missing values used to solve classification problems or regression problems	High space complexity High storage resources for classification
Logistic regression model	simple Classification has very small computation and low storage resources highly interpretable	It is easy to underfit, and the general accuracy is not too high Does not handle a large number of multi-class features or variables well For nonlinear features, a transformation is required Logistic regression does not perform very well when the feature space is large

In this study, logistic regression analysis helped us screen out seven independent risk factors for BM in NSCLC. And, through various ML algorithm verification, it is found that all features have essential contributions in the process of predicting BM, which is in high agreement with the logistic regression analysis.

In previous studies (34–36,) T stage, N stage and grade are considered risk factors for BM of cancer. Jie et al. found that (37) patients are more likely to have metastases, if they had higher N stage at diagnoses. In this research, we also found that lymphatic metastasis promotes BM in NSCLC patients and with increasing N stage, the risk of BM in NSCLC patients also increases. Fan et al. (35) found that T stage and tumor grade affected BM of renal cell carcinoma. In this research, we also found that T stage and tumor grade play an important role in predicting the development of BM in NSCLC patients. And the BM of NSCLC was related to the higher tumor grade and advanced T stage.

Interestingly, BM in NSCLC are more likely to occur in patients who have been treated with chemotherapy drugs in this study, which may be due to the misuse of chemotherapy drugs, resulting in their toxic effects on normal cells and promoting the proliferation of tumor cells (38, 39). Hui et al. (40) found that adenocarcinoma was related to a high risk of BM. In this study, we found that tumor histological type is an important character that affects BM. And adenocarcinoma, the most common tumor type of NSCLC, is more prone to BM than others. In addition, sex and the laterality of the primary tumor site can also affect BM of NSCLC patients. Many studies have shown that gender affects the development of tumors (41, 42). In this study, we found that male at a higher risk of bone metastases than females. Moreover, we found that left primary lung cancer is more likely to have bone metastases than right, which may be associated with the left lung being close to the heart, leading to more hematogenous metastasis of the left lung tumor.

In this study, we constructed a predictor based XGB algorithm with SEER data to predict BM in NSCLC. This research can help clinicians make better clinical decisions and promote the integration of medicine and machine learning. Meanwhile, this study has some limitations. First, Current machine learning is almost entirely statistical or black-box, bring severe theoretical limitations to its performance (43). Second, we cannot comment on which chemotherapeutic agents affect NSCLC BM because the SEER database does not record the treatment regimen and dosage of chemotherapy patients. Third, most of the variables in SEER database are clinical, which limits the accuracy of model prediction to some extent. Fourth, important parameters closely related to lung cancer such as dust, smoking, passive smokers, Tobacco chewing, and alcohol are missing in the SEER database, resulting in the failure of these parameters to be included in the predictor. In the future, with the continuous improvement of the database, we will incorporate more correlation parameters associated with the BM of NSCLC into the web predictor to improve its adaptability.

In conclusion, in this study, we found that XGB algorithm performed best in six different algorithms and then as a tool build a web predictor for predicting BM of NSCLS which was accurate, simple and convenient to operate. This web predictor can predict BM of NSCLS easily and assist clinicians in diagnosis and making better clinical decisions for NSCLS patients.

## Data availability statement

The datasets presented in this study can be found in online repositories. The names of the repository/repositories and accession number(s) can be found in the article/supplementary material.

## Ethics statement

We received permission to access the research data file in the SEER program from the National Cancer Institute, US. Approval was waived by the local ethics committee, as SEER data is publicly available and de-identified. This study was approved by the Ethics Committee of the First Affiliated Hospital of Nanchang University, and cases from the First Affiliated Hospital of Nanchang University signed a written informed consent form. The patients/participants provided their written informed consent to participate in this study. Written informed consent was obtained from the individual(s), and minor(s)’ legal guardian/next of kin, for the publication of any potentially identifiable images or data included in this article.

## Author contributions

MPL and WCL conceived of and designed the study. MPL, WCL, BLS, and NSZ performed analysis and generated the figures and tables. MPL and WCL wrote the manuscript, and ZLL, SHH, ZHZ and JML critically reviewed the manuscript. All authors contributed to the article and approved the submitted version.
